# Garden and Landscape-Scale Correlates of Moths of Differing Conservation Status: Significant Effects of Urbanization and Habitat Diversity

**DOI:** 10.1371/journal.pone.0086925

**Published:** 2014-01-27

**Authors:** Adam J. Bates, Jon P. Sadler, Dave Grundy, Norman Lowe, George Davis, David Baker, Malcolm Bridge, Roger Freestone, David Gardner, Chris Gibson, Robin Hemming, Stephen Howarth, Steve Orridge, Mark Shaw, Tom Tams, Heather Young

**Affiliations:** 1 The University of Birmingham, Birmingham, United Kingdom; 2 The Garden Moth Scheme, Birmingham, United Kingdom; University of Northampton, United Kingdom

## Abstract

Moths are abundant and ubiquitous in vegetated terrestrial environments and are pollinators, important herbivores of wild plants, and food for birds, bats and rodents. In recent years, many once abundant and widespread species have shown sharp declines that have been cited by some as indicative of a widespread insect biodiversity crisis. Likely causes of these declines include agricultural intensification, light pollution, climate change, and urbanization; however, the real underlying cause(s) is still open to conjecture. We used data collected from the citizen science Garden Moth Scheme (GMS) to explore the spatial association between the abundance of 195 widespread British species of moth, and garden habitat and landscape features, to see if spatial habitat and landscape associations varied for species of differing conservation status. We found that associations with habitat and landscape composition were species-specific, but that there were consistent trends in species richness and total moth abundance. Gardens with more diverse and extensive microhabitats were associated with higher species richness and moth abundance; gardens near to the coast were associated with higher richness and moth abundance; and gardens in more urbanized locations were associated with lower species richness and moth abundance. The same trends were also found for species classified as increasing, declining and vulnerable under IUCN (World Conservation Union) criteria. However, vulnerable species were more strongly negatively affected by urbanization than increasing species. Two hypotheses are proposed to explain this observation: (1) that the underlying factors causing declines in vulnerable species (e.g., possibilities include fragmentation, habitat deterioration, agrochemical pollution) across Britain are the same in urban areas, but that these deleterious effects are more intense in urban areas; and/or (2) that urban areas can act as ecological traps for some vulnerable species of moth, the light drawing them in from the surrounding landscape into sub-optimal urban habitats.

## Introduction

The most common and widespread species are likely to play the most important role in supporting ecosystem function and services, but worryingly many of these species have been shown to be in decline [1], [2], [3], [4]. Moths are ubiquitous in vegetated terrestrial environments [5] and are: known pollinators of many species of plant [6], [7], important herbivores of crops and wild plants [8], [9], and food for numerous species of rodents, birds, and bats [8], [10], [11], [12]. However, populations of many common and widespread macro-moths have declined in the UK [13], [14], Finland [15] and the Netherlands [16] in recent decades, and these declines are likely to be representative of the fortunes of moths in highly developed landscapes in other countries. Reasons suggested for these declines include habitat loss and fragmentation due to the intensification of agriculture and forestry, light pollution, climate change, urbanization, agro-chemical pollution, and soil nitrogen enrichment due to air pollution [4], [5], [13], [14], [17], [18]; but there is as yet, little evidence to indicate which factor, or combination of factors, are driving these declines.

Some of the suggested reasons for declines in moth numbers occur over long time-scales, and can, with caution, be investigated using space-for-time approaches [19], [20]. For example, bioclimate models based on current species occurrence across gradients in temperature, can be used to predict the effect of future climate change on the abundance of that species [21], [22]. Alternatively, the spatial distribution of species across gradients of agricultural and urban development could be used to indicate the likely responses of this species to future landscape developments, or to infer past changes. Studies have illustrated the utility of moth assemblages as indicators of the effects of habitat degradation, habitat fragmentation [23], [24], [25], and climate change [12], [26], [27]. Highly developed landscapes, such as those of the UK, characteristically encompass a patchwork of small, highly fragmented patches of semi-natural or favourably managed habitat set within a matrix of intensively managed agricultural and urbanized areas. Within this landscape, gardens can provide substantial habitat resource [28], [29], [30], [31], especially for highly mobile species able to utilise resources from spatially fragmented habitats [32], [33]. In these complex working landscapes, multiple potential anthropogenic threats operate simultaneously on moth assemblages at a variety of spatiotemporal scales. Analysis of garden and landscape-scale relationships with moth assemblages has the potential to aid the understanding of moth declines, but trends are likely to be complex, interconnected and nuanced, requiring extensive data-analyses to successfully identify patterns.

The Garden Moth Scheme [34] is a citizen science project that began collecting data from moth light traps in the West Midlands region of the UK in 2003 and in 2007 expanded to include the whole of the UK (including the Channel Islands) and Ireland. Data gathered on the scheme were analysed as part of the Open Air Laboratories (OPAL) project [35], [36], [37]. In 2010 there were 314 participants recording 195 species across all study regions for a target of 36 weeks per year, to provide around 21,000 hours of recorder effort. Participants record habitat features in their garden and wider-scale landscape variables thought likely to influence moth assemblages [38]. This combination of carefully sampled data on moth assemblage, and local and landscape scale variation in habitat makes the data gathered through the scheme well-suited to the investigation of factors controlling moth assemblage in a highly developed landscape, and to thereby postulate reasons for recent declines.

This document explores British GMS data collected during 2010, analysing the effects of garden habitat and landscape-scale variation on the diversity and abundance of moths. It also uses the conservation status classification of Conrad et al. [13] to investigate whether relationships between habitat and assemblage are the same for species that are known to be declining or increasing in abundance. It specifically asks the following research questions:

1. Which garden habitat and landscape-scale features (e.g. urbanization intensity, proximity to coast, proximity to woodland) most strongly influence the species richness, total abundance, and the abundance of individual species of moth?

2. Do these spatial habitat and landscape associations differ for species that are declining or increasing in abundance?

## Methods

### Data Extent and Quality

**Figure 1:**
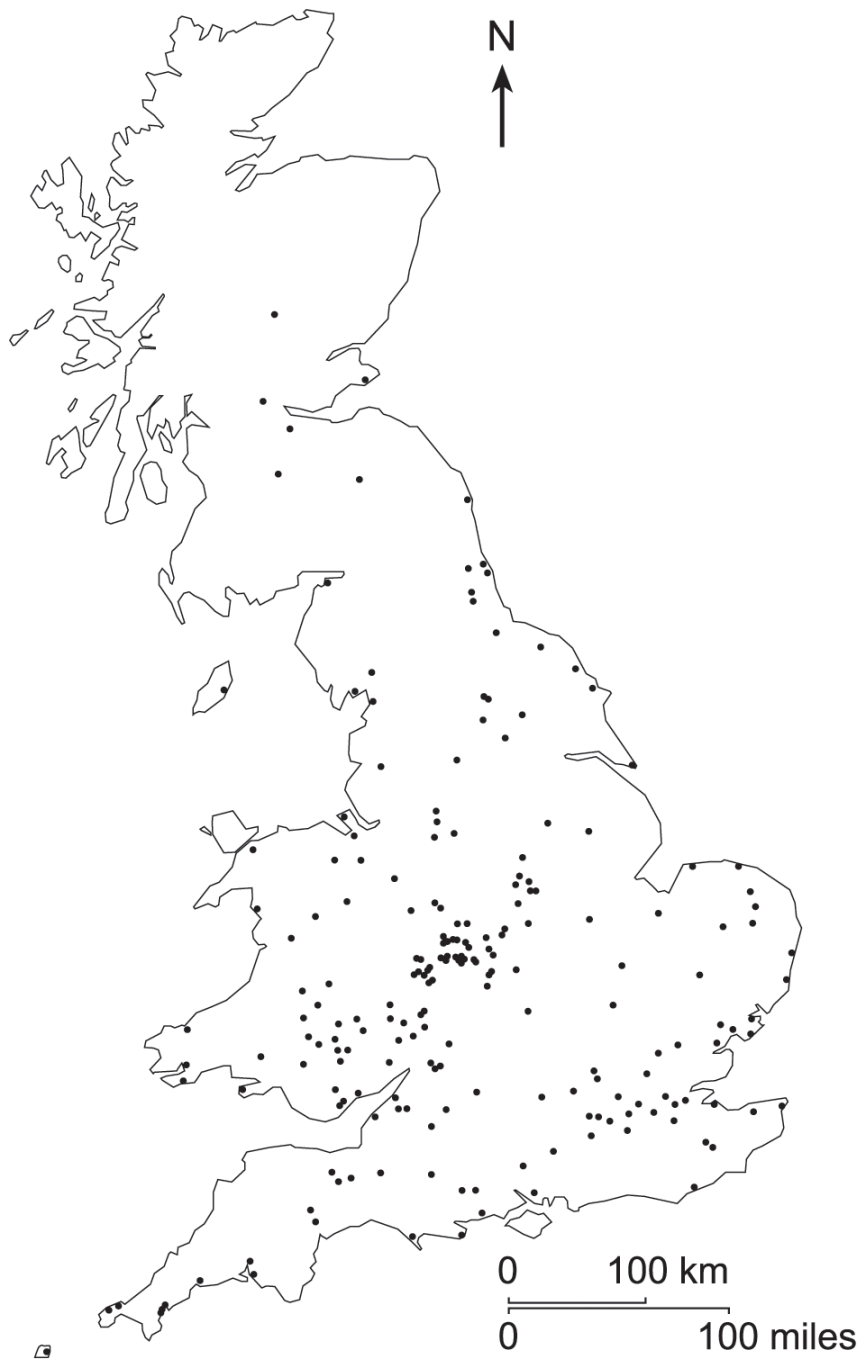
The spatial distribution of the 214 sample sites used in analyses.

This document expands on the analysis of GMS data from 2010 used in Bates et al. [38] to assess the effect of trap and bulb type on moth catch. The full dataset was rationalised to remove explanatory variable combinations with small numbers of observations, and datasets with deficient number and temporal distribution of samples. The final dataset contained 214 sites distributed across England, Wales and Scotland (Figure 1). The GMS focuses on species easily identified when alive using readily available identification guides such as [39]. Each regional coordinator checks submitted data for unusual records, taking into account rarity, phenology and distribution, and data are further checked by the national coordinators. Unusual records are queried with the participant, and if found to be unsupported by photographs or visual confirmation from a volunteer expert, are removed from the database. Within each survey period identification training is supported using a GMS on-line forum where participants can post photographs, with more experienced participants guiding new participants to further improve identification reliability. Most species are ‘macro’ moths, but some easily identified ‘micro’ moths are included (Table S1).

### Moth Sampling

The target sampling program was on Friday each week for 36 weeks from March to November. Sampling on the Friday was not always possible, in which case participants could sample up to three days early or late providing that they did not sample on successive nights, and did not ‘cherry pick’ the best nights in terms of weather. Participants could be taken ill or take a holiday, so data from a minimum of 31 weeks were used, with no gaps in sampling greater than three weeks over the whole sampling period, or no sampling gaps greater than two weeks during June to September, when moths were most abundant in 2010. Participants were required to: sample for all hours of darkness; check the traps as early as possible after dawn to reduce predation; include moths resting in the immediate surroundings of the trap; and record events when a trap was run, but no moths sampled. Participants that submit information on two or more traps have to make sure that they are separated by at least 50 m or a large light-proof object (e.g. a house).

Moths were sampled using two types of light trap, Skinner and Robinson [40]; and six categories of bulb, 15W actinic (low pressure fluorescent tubes), 20–40W actinic, 60W actinic, 80W mercury vapour (high pressure mercury blended filament), 125W mercury vapour and 160W blended (equivalent of 80W mercury vapour and 80W tungsten filament incandescent bulbs). Both mercury vapour and actinic bulbs produce a proportion of their output as the UVA radiation most effective at attracting moths; tungsten filaments produce their light in the less effective, visible part of the spectrum [38], [41].

### Environmental Variables

Participants provide the following details by questionnaire: grid reference; trap and bulb type; soil acidity (acidic, neutral or basic); distance to the nearest field (i.e. agricultural); distance to nearest woodland; distance to nearest water; distance to nearest streetlight (all distances = adjacent, <50 m, 50 m–2 km, >2 km); size of garden (<50 m^2^, 50–200 m^2^, 200–400 m^2^, >400 m^2^); and presence of the following garden microhabitats: lawn >25 m^2^, log pile, pond, tree >10 m, oak tree >10 m, compost heap, long grass, native species hedgerow, wildflower meadow, Honeysuckle (*Lonicera periclymenum* L.), Ivy (*Hedera helix* L.), pussy willow (flowering *Salix* spp.), Common Nettle (*Urtica dioica* L.) patch, and Butterfly-bush (*Buddleja davidii* Franch.).

UK national grid references were converted to latitude and longitude in decimal degrees to provide continuous numeric figures for spatial location. The altitude of each sample site was measured using Google Earth. Sample sites were categorised as either urbanized (urban or suburban) or rural by AJB using Google Earth. Rural sites were considered those that were in countryside (fields, nature reserves or woodland) or villages less than 1×1 km (by total area). Sites were classified as coastal if they were within 2 km of the high tide mark. The presence of most garden habitat features were strongly collinear, so these were summed to give one ‘garden microhabitats’ variable. The explanatory variables used in analyses are shown in Table 1.

**Table 1:** Explanatory variables used in the analyses (act = actinic, MV = mercury vapour).

Variable	Type	Levels	Used in GAMM as
Trap type	nominal	Skinner & Robinson	Random factor
Bulb type	nominal	15W act, 20–40W act, 60W act, 80W MV, 125W MV, 160W blended	Random factor, nested within trap
Altitude	continuous, m asl	–	Fixed factor
Garden microhabitats	continuous, count	–	Fixed factor
Garden size	nominal	>50 m^2^, 50–200 m^2^, 200–400 m^2^, >400 m^2^	Fixed factor
Latitude & Longitude	continuous, decimal degrees	–	Smoothing spline
Soil type	nominal	Acid, Neutral & Basic	Fixed factor
Urbanization	nominal	Urbanized & Rural	Fixed factor
Distance to field	nominal	Adjacent, <50 m, 50 m–2 km, >2 km	Fixed factor
Distance to streetlight	nominal	Adjacent, <50 m, 50 m–2 km, >2 km	Fixed factor
Distance to wood	nominal	Adjacent, <50 m, 50 m–2 km, >2 km	Fixed factor
Distance to water	nominal	Adjacent, <50 m, 50 m–2 km, >2 km	Fixed factor
Distance to coast	nominal	0–2 km, >2 km	Fixed factor

### Conservation Status

Conrad et al. [13] analysed a 35-year dataset of abundances of species of British macro-moth and classified species, based on IUCN criterion, as ‘increasing’ (change rate >0 10yr^−1^), ‘declining’ (change rate 0–30% 10yr^−1^ decline), ‘vulnerable’ (>30% 10yr^−1^ decline), and ‘endangered’ (>50% 10yr^−1^ decline). The vulnerable and endangered species are part of a UK Biodiversity Action Plan (BAP) species list reviewed in 2007, termed ‘common and widespread, but rapidly declining moths – research only’[42]. The GMS dataset analysed did not contain any species classified as endangered by Conrad et al. [13], but analysed the total abundance and species richness of moths in each of the categories: increasing, declining and vulnerable (Table S1).

### Data Analysis

In-flight moth abundance and light trap efficiency are known to vary markedly from one night to the next due to changes in air temperature, wind speed, cloud cover and lunar phase [41], [43]. To time-average this un-parameterised variation, species counts from the 2010 data were summed.

Between-site comparisons of species richness can be misleading when the total number of individuals sampled at each site varies, because as more individuals are sampled, more species are likely to be recorded [44]. Therefore measured species richness figures were supplemented by estimates of total species richness for each site using the Chao2 non-parametric extrapolation method [45], [46]. Nonparametric estimators of asymptotic species richness have been the most successful estimators, and Chao2 was selected as the sample-based estimator because the exploration of rarefaction curves suggested that a large proportion of the target moth assemblage had been sampled ([47], Nick Gotelli pers. comm.).

We opted for a Generalized Additive Mixed Modelling (GAMM) [48] approach as it does not force a parametric relationship between the response and predictor and can deal with non-linearity in response/covariate relationships. As trap design and bulb are known to significantly affect moth catch [38], [41], these variables were used as random factors in analyses [49], with bulb nested within trap, and the remaining covariates as fixed factors. We included a spatial smoothing spline using latitude and longitude to account for larger scale variability related to site location [50]. Response variables measured were total observed species richness (Sobs); estimated total species richness (Chao2); total abundance; the abundance of individual species (for which valid models could be fitted); the richness of increasing, declining and vulnerable species; and the abundance of increasing, declining and vulnerable species; with the fixed effect explanatory variables shown in Table 1.

As data were over-dispersed, a negative binomial distribution was used in the GAMM [51]. Competing model fit and parsimony were assessed using small sample unbiased Akaike information criterion (AICc) to generate sets of competing models [52], [53]. Ninety five percent confidence interval set of models were created based on calculated Akaike weights including the ‘best’ (lowest AICc) and competing models. Parameter estimates and adjusted R^2^ values within these confidence sets were model averaged using calculated Akaike weights [52], [53]. GAMM was implemented in Brodgar v2.7.2 [54], which is a user interface that relies heavily on the freeware R v2.9.1 [55].

During analyses it became clear that the effect of urbanization differed for species categorised as increasing, and species categorised as declining, despite both groups showing an overall negative relationship with urbanization intensity. This varying response of species of different conservation status [13] was explored using ordination in Canoco for Windows version 4.51 [56] with the three indicators of urbanization: urbanization, distance to field and distance to street light used as explanatory variables. The gradient lengths from initial indirect ordinations using detrended correspondence analysis (DCA) were all short (<3) so redundancy analysis (RDA) was selected as the most appropriate ordination method [57]. Scaling focused on inter-species correlations and species scores divided by their standard deviation were used for RDAs. Model significance values were generated using Monte Carlo analyses (9999 permutations, with a random seed).

### Light Competition

Light pollution (or other light sources, e.g. moonlight) by raising ambient light levels can decrease the efficiency of moth light traps by reducing the relative difference in light intensity between the trap and its surrounds, thereby reducing the trap’s area of effect [41], [43]. Greater levels of light pollution in urbanized habitats could reduce the efficiency of light traps compared to rural sites, thereby creating an observed reduction in moth richness that is a sampling artefact, rather than a real reflection of the population richness. The existence of this sampling artefact was tested for by calculating a proportional indicator of sample ‘completeness’ by dividing the observed species richness (Sobs) by the estimated total species richness (Chao2). This measure was used as a response variable in a GAMM structured as above with the same initial explanatory variables but with a Gaussian distribution to test for an effect of urbanization (associated with greater levels of light pollution) on sample completeness.

### GMS Data Access

GMS data are stored by the GMS and are freely available to researchers contacting the GMS, following the completion of a data supply and use agreement.

## Results

A total of 385,870 individual moths were sampled in the dataset. Minimum, average, and maximum total moth abundances, measured species richness (Sobs), estimated total species richness (Chao2), abundance of each conservation status group, and richness of each conservation group, per sample site are shown in Table 2. Only three sample sites had Chao2 estimates greater than the maximum ‘real’ species richness of 195, suggesting that this estimator was generally performing well.

**Table 2:** The minimum, mean and maximum values per sample site of response variables used in analyses.

	Minimum	Mean	Maximum
Total abundance	171	1803	6129
Special richness (Sobs)	45	112	187
Estimated species richness (Chao2)	60	138	223
Increasing species abundance	65	743	3236
Declining species abundance	45	686	2558
Vulnerable species abundance	0	91	538
Increasing species richness	11	30	46
Declining species richness	17	51	85
Vulnerable species richness	0	11	20

**Figure 2:**
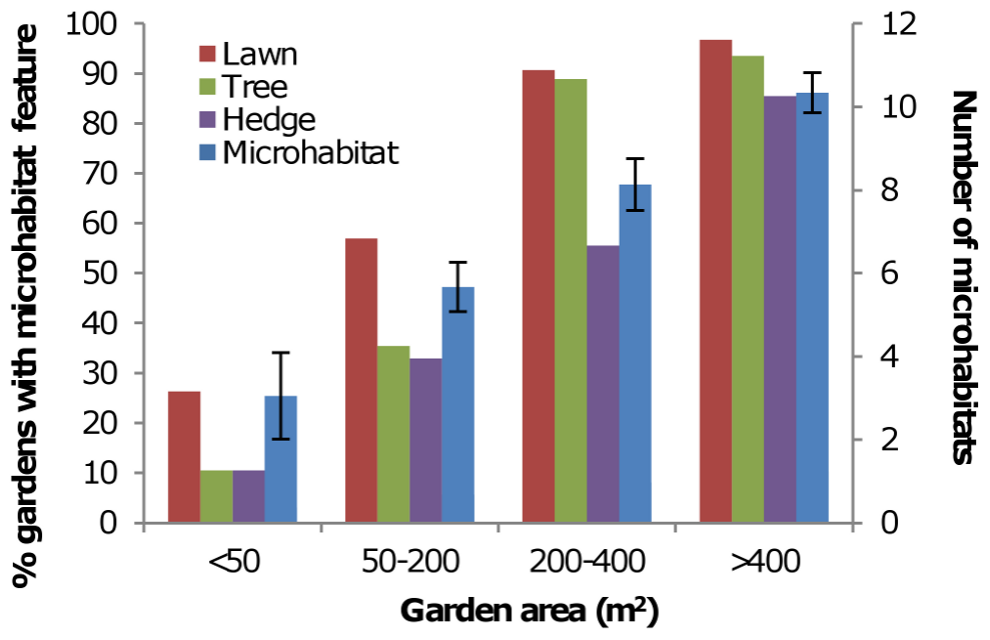
Relationship between garden area and the total number of microhabitats and percentage occurrence of three key microhabitat features: lawn, tree and hedge. Error bars +/−95% confidence intervals.

**Figure 3:**
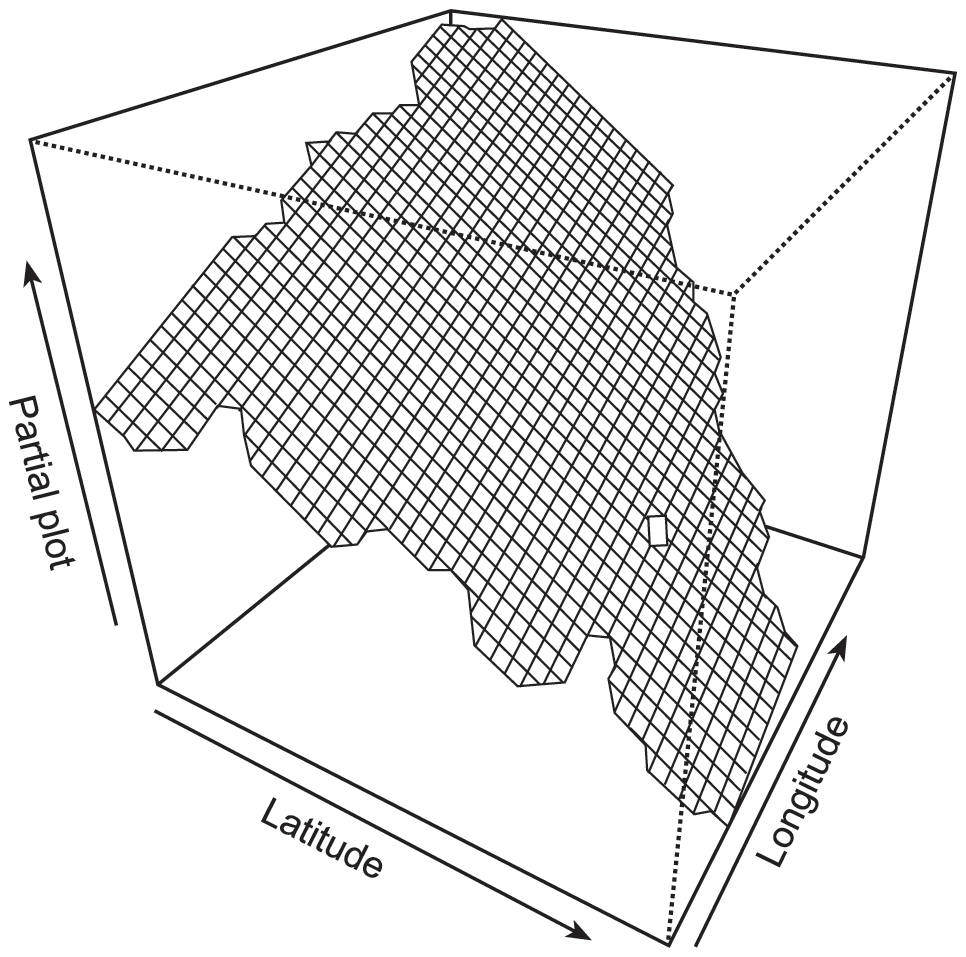
Example partial plot visualisation of the Latitude Longitude smoother used in the GAMM with total moth abundance as the response variable. Total abundance was highest in the SE of Britain (compare against map of Britain in Figure 1).

Model averaged GAMMs of species richness, estimated richness and total abundance showed similar relationships (Table 3). All were negatively associated with increased levels of urbanization. For species richness this was shown by a relatively weak (+7%) but significantly higher species richness in rural compared to urbanized sample sites, and a stronger (−37%) effect of distance to field. The latter variable showed little difference between sites that were adjacent and <50 m away from fields, and then an increasing difference between these sites and sites that were 50 m−2 km and >2 km away from fields. This break point in distance to field was similar for estimated richness and total abundance, with significant negative effects observed once a site was 50 m–2 km away from fields, which was strongest once a site was >2 km from fields. For total abundance, this effect was particularly strong, with 65% less individuals at sites >2 km from fields (i.e. >2 km into a town or city) than those adjacent to fields (i.e. in rural areas). For total abundance, distance from coastline was also a significant explanatory variable, with 26% less individuals at sites >2 km from the coast (Table 3). Garden size was significantly positively associated with richness, estimated richness and total abundance (Table 3), with gardens 50 to more than 400 m^2^ higher than gardens <50 m^2^. Garden size as a variable was quite strongly associated with the occurrence of some microhabitats and total microhabitats (Figure 2). Species richness, estimated richness and total abundance all showed significant spatial patterns, with all three variables highest in the south east of Britain, as shown for total abundance (Figure 3).

**Table 3:** Model averaged GAMMs of explanatory variable effects for species richness, estimated species richness, total abundance, and index of sample completeness.

	Adj. R^2^	Lat. Long.	Micro.	Alt.(100 m)	Urban.2	pH2	pH3	D. field2	D. field3	D. field4	D. wood2	D. wood3	D. wood4	Coast.2	G. size2	G. size3	G. size4
Richness (Sobs)	0.287	P<0.01highest in SE	0.000	0.000	**0.074**	0.000	0.001	0.030	−**0.125**	−**0.368**	0.000	−0.002	−0.002	0.008	**0.207**	**0.119**	**0.171**
Estimated richness (Chao2)	0.243	P<0.05 in 7of 8 modelshighest in SE	0.000	−0.012	0.017	0.000	0.000	0.040	−**0.066**	−**0.309**	–	–	–	–	**0.151**	0.055	**0.116**
Tot. abun.	0.227	P<0.001highest in SE	0.005	–	0.114	–	–	−0.049	−**0.374**	−**0.648**	−0.038	−0.099	0.091	−**0.258**	**0.467**	**0.396**	**0.489**
Sobs/Chao2	0.148	NS	–	–	**0.065**	–	–	–	–	–	–	–	–	–	–	–	–

Parameters with numbers were included in the model set. Parameters in bold are those that were significant at *P*<0.05 for at least one model in the model sets. Reported values of nominal explanatory variables are for parameter effects relative to the first level of that parameter; so for example for species richness, D.field2 (<50 m) is 3% higher than D.field1 (adjacent), and D.field4 (>2 km) is 37% lower than D.field1 (adjacent). Values for the latitude longitude smoothing spline are the *P* values of the spline, together with a description of the effect. Abbreviations are: Tot. abund. = total abundance, Sobs/Chao2 = sample completeness, Adj. R^2^ = adjusted R^2^, Lat. Long. = latitude longitude, Alt. = altitude, Urban. = urbanization, D. field = distance to field, D.wood = distance to wood, Coast = distance to coast, G.size = garden size, SE = south east, and NS = not significant.

**Figure 4:**
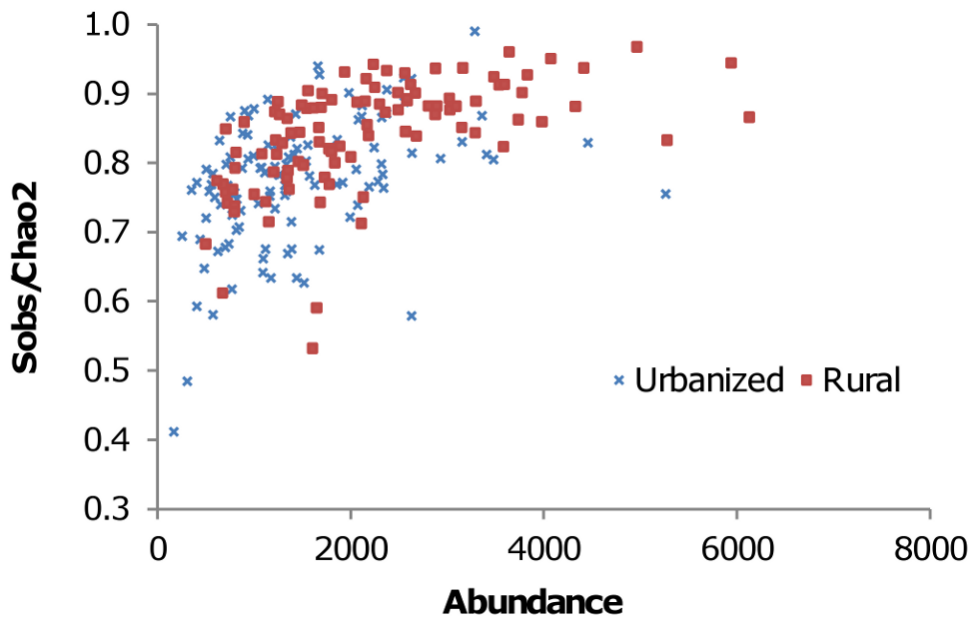
Species richness sample ‘completeness’ (observed number of species Sobs/Chao2 predicted number of total species) in urbanized and rural sites of varying total moth abundance.

Sample completeness was significantly positively associated with rural sites, with rural sites 6.5% more complete than those in urbanized sites (Table 3); it was also related to abundance at the sites (Figure 4). It is worth noting that the associations with indicators of urbanization intensity (urbanization and distance to field) were less strong for estimated species richness than for measured species richness.

GAMMs for 14 of the more abundant of the 195 species are shown in Table 4. The patterns overall were similar to those for the summary richness and abundance variables, namely: (1) more species were significantly positively associated with southern than northern locations; (2) more species were significantly positively associated with coastal than inland locations; (3) there were few strong relationships with altitude, soil pH, proximity of woodland, or proximity of water; (4) the effect of more garden microhabitats or larger gardens when significant, was almost always positive (except for a weak negative relationship for *Peribatodes rhomboidaria*); and (5) there were more species negatively associated with urbanization (urbanization, distance to field and distance to street light) than were positively so. However, despite the broad trends, associations with the explanatory variables were species specific, with, for example, some species more abundant in the north and west of the country (e.g. *Chloroclysta truncata* and *Noctua pronuba*). In particular, of the twelve abundant GAMM-analysed species that showed significant relationships with the urbanization variables, eight showed negative relationships, but four showed positive relationships.

**Table 4:** Model averaged GAMMs of explanatory variable effects for fourteen species of moth.

	*Eurrhypara hortulata C*	*Chloroclysta truncata G*	*Idaea aversata G*	*Peribatodes rhomboidaria G*	*Agrotis exclamationis N*	*Apamea monoglypha N*	*Axylia putris N*	*Noctua comes N*	*N. pronuba N*	*Orthosia cerasi N*	*O. gothica N*	*Ochropleura plecta N*	*Orthosia incerta N*	*Xestia c-nigrum N*
Adj. R^2^	0.089	0.132	0.210	0.142	0.201	0.267	0.120	0.224	0.279	0.311	0.267	0.415	0.333	0.184
Lat. Long.	P<0.001 highest in S & central areas	P<0.001 highest in W	P<0.001 highest in S	P<0.001 highest in S & central areas	P<0.001 highest in S	P<0.001 highest in N & E	P<0.001 highest in SW	P<0.001 highest in N & E	P<0.001 highest in N of England	P<0.05 small-scale trends	NS	P<0.001 highest in SE	P<0.001 highest in N	P<0.001 highest in S, particularly SE
Micro	0.009		0.001	**−0.030**			**0.060**	**−**0.001	**0.030**	**0.040**		**0.033**	**0.048**	
Alt. (100 m)	**−**0.160	**0.277**	0.000	**−**0.342	**−0.199**	**0.249**	0.000	0.000	0.108	0.000	0.000	0.000	**−**0.026	**−1.109**
Urban.2	**0.364**	**−**0.013	0.001	**−**0.039		0.015	**0.471**	**−**0.229		0.011	**0.481**	**0.473**	**0.501**	
pH2			0.231	**0.390**	**0.040**	0.001			0.199				**−**0.100	**0.050**
pH3			**0.505**	0.368	0.040	**−**0.311			0.097				**−**0.442	0.042
D.field2		**0.435**	**0.411**	**0.405**		0.026	**−**0.238	**0.330**		0.236	**−**0.110	**−**0.087	**−**0.124	**−**0.315
D.field3		**0.365**	**0.302**	**0.320**		**−0.318**	**−0.452**	**−**0.028		**−0.432**	**−0.651**	**−0.489**	**−0.431**	**−0.923**
D.field4		**1.012**	0.153	0.150		**−1.100**	**−0.968**	0.129		**−1.033**	**−1.024**	**−1.499**	**−1.694**	**−1.948**
D.wood2					**0.609**	0.057	0.217	0.003		**−0.681**	**−**0.326		0.001	0.129
D.wood3					**0.246**	0.150	0.065	0.212		**−0.629**	**−0.371**		**−**0.018	0.067
D.wood4					**0.529**	**0.731**	0.161	**0.560**		**−0.779**	**−**0.132		**−**0.012	0.112
D.wat.2		0.241	0.024		0.002	0.052	**−**0.247		0.109	**−**0.060		**−**0.082		**−**0.075
D.wat.3		0.092	0.003		**−**0.046	0.050	**−**0.244		0.112	0.176		**−0.437**		0.038
D.wat.4		0.341	0.008		**−**0.009	0.048	0.257		**−**0.065	0.287		**−**0.004		**0.541**
Coast.2	**−0.520**	**−**0.074		0.172	**−0.387**	**−0.831**		**−0.323**	**−0.554**	**0.350**	**−**0.022	0.040	**0.643**	
D.s.light2		0.214	0.140				0.153	0.034					0.043	**−0.363**
D.s.light3		0.089	**−**0.163				**−**0.116	**−0.350**					**0.645**	0.353
D.s.light4		**−0.649**	**−0.646**				**−**0.531	**−0.839**					0.332	0.147
G.size2				0.023	**0.394**	**0.785**			**0.182**		**0.688**	0.140		
G.size3				0.019	**0.591**	**0.608**			**0.198**		**0.615**	**0.206**		
G.size4				0.015	0.275	**0.445**			**0.201**		**1.119**	**0.211**		

Parameters in bold are those that were significant at *P*<0.05 for at least one model in the model sets. Values for the latitude longitude smoothing spline are the *P* values of the spline, together with a description of the effect. Abbreviations are: C = Crambidae, G = Geometridae, N = Noctuidae, Adj. R^2^ = adjusted R^2^, Lat. Long. = latitude longitude, Micro. = garden microhabitats, Alt. = altitude, Urban. = urbanization, D. field = distance to field, D.wood = distance to wood, D.wat = distance to water, Coast = distance to coast, D.s.light = distance to street light, G.size = garden size, N E S W = north east south west, and NS = not significant.

**Figure 5:**
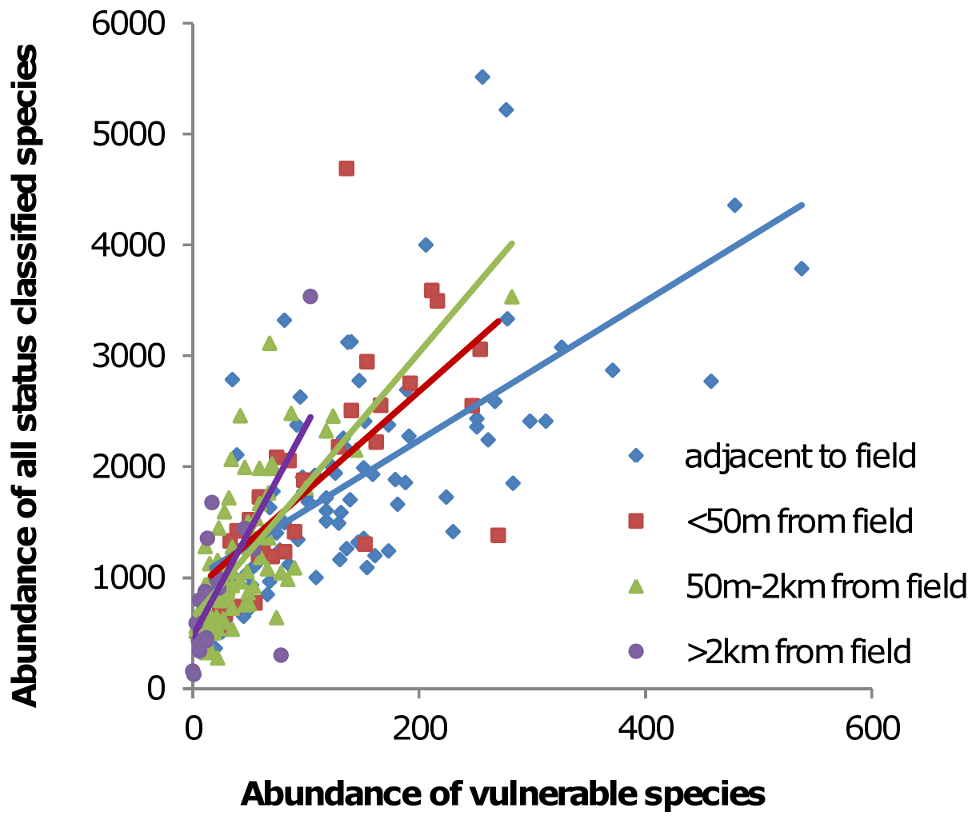
Example relationship between the abundance of all status classified species (sum of increasing, decreasing, and vulnerable) and the abundance of vulnerable species. There was a strong overall positive relationship. However, plotting and fitting linear regression lines to sites of differing distance to field showed a distinct difference in the abundance of vulnerable species. At sites >2 km away from fields (sites in towns and cities) there was a lower proportion of vulnerable species than at sites adjacent to fields (rural sites).

**Figure 6:**
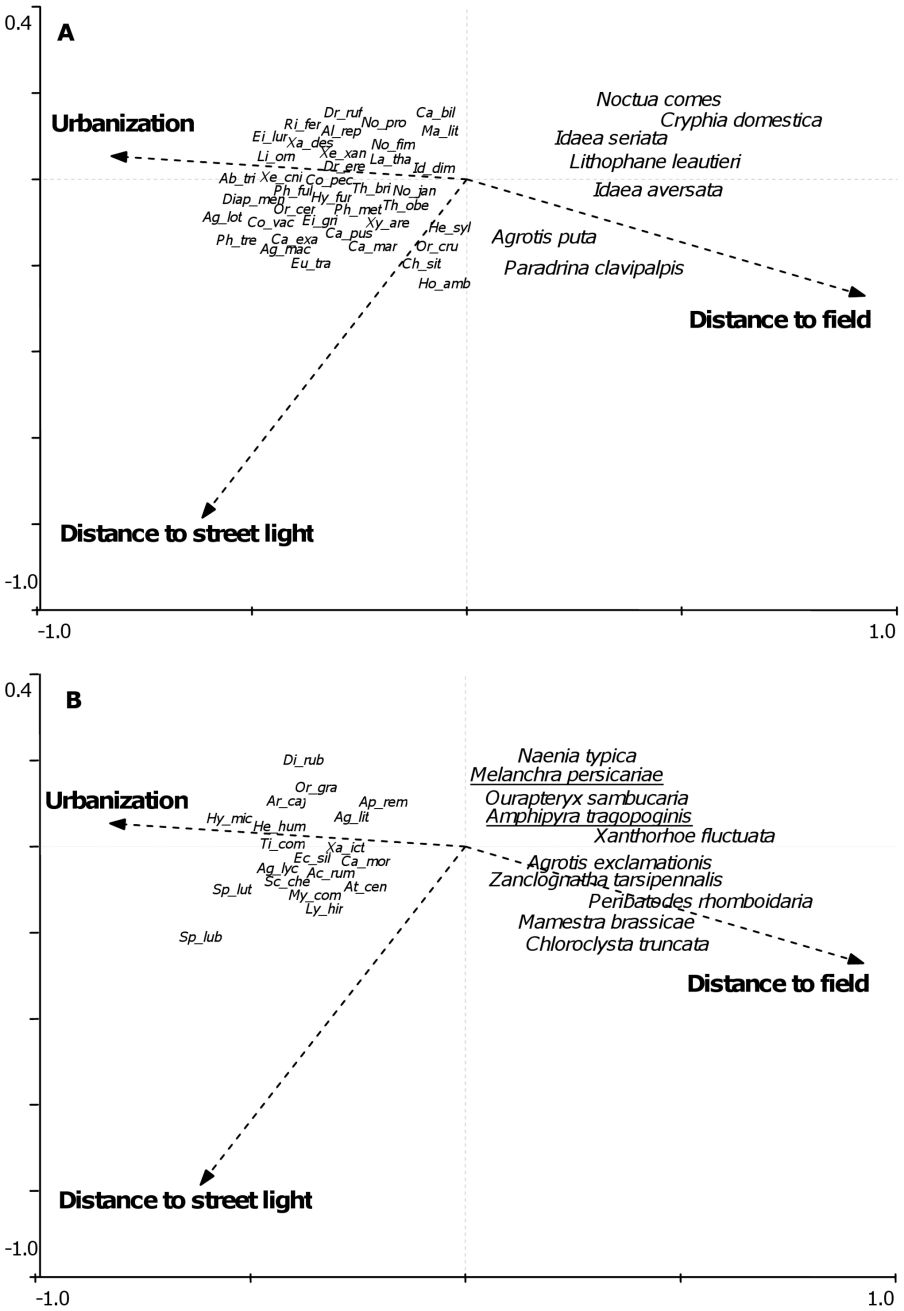
RDA ordination plot of species abundances in relation to explanatory variables describing urbanization level. Species associated with higher levels of urbanization are situated towards the top right of the two panels. Panel A shows increasing species, panel B shows vulnerable species (abbreviated species names and full species name underlined) and declining species associated with higher levels of urbanization (full species names, not underlined).

GAMMs for the richness and abundance of increasing, declining and vulnerable species largely showed the same associations as total richness and abundance so are not shown. However, the data suggested that despite the strong positive relationship between the richness and abundance of moths in each status category and total richness and abundance, the relative proportion of vulnerable and increasing species differed with level of urbanization (Figure 5). Figure 6 and Table 5 show the RDA analysis illustrating how status classified species were associated with the three indicators of urbanization intensity: urbanization, distance to field, and distance to street light. Increasing, declining and vulnerable species were all predominately negatively affected by urbanization intensity, but for all three groups the response to urbanization was species specific, with some species positively associated with higher levels of urbanization. However, the overall response was slightly different between increasing (Figure 6A) and vulnerable species (Figure 6B). Of the vulnerable species, only 9.5% showed a positive associated with urbanization. This figure was 14.9% for increasing species, and increasing species were generally less strongly negatively associated with urbanization in comparison (Figure 6).

**Table 5:** Eigenvalues, species-environment correlations, cumulative percentage variance in species data explained, and significance of the first and all canonical axes in the RDA.

	Axis 1	Axis 2
**Eigenvalues**	0.053	0.006
**Species-environment correlations**	0.491	0.197
**Cumulative % variance of species data**	5.3	5.9
**Significance of first canonical axis**	*F* ratio = 11.840	*P* = >0.001
**Significance of all canonical axies**	*F* ratio = 4.622	*P* = >0.001

## Discussion

### The Importance of Local Habitat Extent and Heterogeneity

Smaller gardens are likely to have smaller trap sampling areas because barriers to light (e.g. walls, hedgerows) will reduce the lateral extent of the lighted area. Therefore, smaller gardens might be associated with lower assemblage richness because of a sampling artefact. However, the estimated species richness should not have been strongly influenced by such a reduced area of effect because the estimates were based on the underlying distribution of species among samples, which is related to, and essentially incorporates sample size. The estimated total richness did show a significant relationship with garden size, suggesting that this was in part a ‘real’ association. However, this relationship was weaker than the relationship with total richness, suggesting that the observed relationship was a combination of sampling artefact and ‘real’ association. The sampling artefact will have most likely have been present in measured total abundance and abundance of individual species, but there is no way of assessing the scale of this bias in the dataset. However, based on the difference between the magnitude of the garden size relationship with estimated and measured species richness, the sampling artefact was smaller (∼35%) than the ‘real’ effect of garden size.

Surrounding the study gardens was a habitat matrix of other gardens, parks, agricultural, woodland and semi-natural habitats, essentially representing a continuous habitat much larger than the garden itself. The moths studied in this analysis all have the ability to easily disperse between the study gardens and other adjacent or nearby suitable habitat, so that, especially for small gardens, the garden will likely only make up partial habitat resources within spatially more extensive habitats [32], [33]. The significant positive associations between garden size and species richness, abundance of individual species, and total abundance are unlikely therefore to be due to habitat patch area effects at the scale of individual gardens. Given the correlation between garden size and the presence and abundance of different garden microhabitats, it is more likely that garden size outperformed the number of garden microhabitats as an explanatory variable in some analyses because garden size incorporated the occurrence of different types of microhabitat *and* some element of the spatial extent of each microhabitat.

Smith et al. [58] found the abundance and diversity of many groups of invertebrates to be positively associated with several elements of garden microhabitat diversity. Of particular relevance, the abundance of moths was positively associated with garden habitat diversity (moth species richness was not measured in this study). The importance of garden microhabitat diversity and extent for supporting larger and more diverse moth assemblages in the current investigation expand the findings of Smith et al. [58], thus strengthening the case for the importance of wildlife gardening for the support of biodiversity and ecosystem functioning [28], [30], [31].

### The Importance of Coastal Habitat

Although species showed varying responses to distance from coast, for the most part, abundance was positively associated with coastal areas. Populations of several of the study species (e.g. *Autographa gamma*, *Hoplodrina ambigua*, *Noctua pronuba*) are immigrants, suspected immigrants, or supplemented by immigration from mainland Europe [39], and therefore might be expected to have larger populations in coastal areas. However, coastal areas of the UK are also associated with a particularly high concentration of rare species of invertebrates associated with, for example, dune systems, salt marshes and eroding cliffs [59], [60]. Inaccessibility, erosion and threat of flooding combine in many coastal areas to create a thin ribbon of habitat relatively protected from intensive agricultural management that is likely to support healthier moth assemblages. The intensification of agriculture is thought to be one of the major causes of farmland biodiversity loss [61], [62], and can reduce moth abundance and species richness relative to land more favourably managed in agri-environment schemes [25], [63]. The degree of agricultural intensification was not measured in the GMS, so the effects of this likely major cause of moth decline cannot be assessed directly using the environmental dataset collected. However, the higher abundance of moths at coastal locations could represent an indirect indicator of the importance of agricultural intensification for the decline of species of common moths.

### The Effect of Light Pollution on Trap Yields

Sites in urban and suburban centres had less complete moth assemblage samples than sites in rural areas, which suggested that greater levels of light pollution associated with urbanization was reducing light trap efficacy. This might be expected, as any increase in the level of ambient night-time light will reduce the relative difference in light intensity between a trap and its surroundings, thereby reducing the traps area of effect [41], [43]. The question might be asked therefore: were the observed significant effects of urbanization intensity on total abundance and species richness merely artefacts of the method used to sample these assemblages? The estimated species richness was also significantly negatively associated with indicators of urbanization, but less strongly than measured species richness. As the measured species richness would have been affected by the light interference bias, this suggests that the urbanization effect on measured species richness was a combination of sampling artefact and ‘real’ effect. This sampling artefact will have most likely been present in measured total abundance and abundance of individual species, but this cannot be directly assessed. However, based on the difference between the magnitude of the urbanization effect between the estimated and measured species richness, the sampling artefact is much smaller (∼25%) than the ‘real’ effect.

### The Importance of Urbanization

In a meta-analysis of invertebrates McKinney [64] found a general trend of reduced species richness in association with urbanization, but that species richness can sometimes be highest in urban or suburban areas. The studies analysed used wide-ranging: focus taxa, methods of urbaninity classification, and sampling design. However, the standardised GLOBENET sampling regime, which focused on carabid beetles, found similarly varying responses of species richness and abundance to urbanization [65], [66], which suggests that invertebrate assemblages responses to urbanization do vary at least in response to urban character, and probably also due to the choice of study taxa.

Most studies of the effects of urbanization on moths, however, have focused on small species pools of relatively poorly dispersing micro moths as bioindicators [67], [68], [69], [70], rather than considering how broader moth assemblages are affected by urbanization, but see [71]. Studies focusing on the effects of urbanization on butterflies, which might be expected to show similar responses to moths given their close taxonomic relatedness and similar habitus, were also reviewed. Some consistent trends emerge from this literature: (1) responses to urbanization are species specific [69], [70], [71], [72], [73], [74], (2) total abundance tends to be negatively associated with urbanization [67], [68], [71], [75], and (3) species diversity tends to be negatively associated with urbanization [67], [71], [72]. Another taxonomic group that might be expected to show similar responses to Lepidoptera given their association with plants and relatively good dispersal ability are bees [76]. Indeed, similar species specific responses to urbanization, with general trends of declining species richness and abundance have been found for bees [77], [78], [79].

In line with most of the relevant published literature, we found that species richness and abundance were negatively affected by urbanization and that individual species responses to urbanization, although usually negative, were positive for some species. The environmental data gathered by the GMS do not allow further differentiation of the effects of the amount or character of built space in the surrounding landscape, but it is clear that urbanization has a strong overall negative effect on moth assemblage. Urbanization is associated with a mixture of many of the factors cited as likely drivers of the decline of common species of moth. Agrochemicals are widely used in gardens and other highly managed urban green spaces [31]. Light pollution levels are higher in urban areas than the surrounding countryside [80], [81], which can potentially increase the disruption of moth navigation, breeding, circadian rhythms and photoperiodism, and increase exposure to predation [17], [82]. Urban landscapes represent an extreme on the continuum of the proportion of unsuitable habitat and habitat fragmentation, and there is growing evidence that the negative effects on insect assemblages are often greater than those associated with agriculture [75], [83].

Van Dyck et al. [4] in a 16-year study of common species of butterfly in the Netherlands showed that total butterfly numbers had declined, but that this decline was species specific, with some species showing increases. Declines were particularly marked in farmland, woodland, and in urban areas. In the current study, moths classified as increasing, declining and vulnerable by Conrad et al. [13] were all negatively affected by urbanization overall. However, interestingly, focused statistical and graphical analyses were able to detect subtle differences in the response of increasing and vulnerable moths to urbanization, with vulnerable moths more strongly negatively affected by urbanization than increasing moths. We propose two hypotheses to explain these differing levels of susceptibility to urbanization: (1) The negative habitat and landscape effects associated with urbanization represent an extreme on the continuum of effects operating throughout the British landscape, and therefore vulnerable species that are declining rapidly throughout the wider landscape will be more strongly affected by the deleterious effects of urbanization because they are responding to the same driving forces. (2) Towns and cities are causing a reduction in moth abundance and diversity in the wider surrounding landscape that extends well-beyond their limited spatial extent, thereby influencing moth numbers throughout Britain. The latter hypothesis in particular warrants further expansion.

Recently, various authors have raised the possibility that urban areas could act as ecological sinks [84] or even ecological traps [85], [86] for a variety of organisms, but particularly birds [83], [87], [88], [89]. For example, van Heezik et al. [89] found that the level of urban domestic cat predation of some species of bird was high enough to make urban populations unsustainable without the supplementation of urban populations with individuals dispersing from surrounding rural habitats. For butterflies, Altermatt [90] recently reported indirect phenological evidence suggesting that many species of day-flying Lepidoptera annually migrate into urban habitat sinks from surrounding agricultural and forested habitats. Levy and Connor [87] found that gardens of insufficient habitat quality and quantity can potentially act as sinks for butterflies. Night flying moths are one of the most likely candidate species-groups for attraction to sub-optimal urban habitat ecological traps, because their attraction to light provides an obvious mechanism by which dispersal to urban areas might be facilitated. Although the unsuccessful use of light traps to locally eradicate pest species of moth, and the persistence of moth populations near to lights suggests that artificial light is unlikely to totally eradicate local moth populations; there exist multiple lines of evidence for significant artificial light induced moth mortality [17], [82]. In addition, as recent research has shown [91], clouds can amplify light pollution so that effects extend for many kilometres outside city boundaries. There therefore exists the potential to draw moths into urbanized areas from wide rural areas, and given that small moth light traps can draw moths from many hundreds of metres away [43], this seems at least feasible for the large domes of light associated with cities. Further research exploring the potential for urban areas to act as sinks or ecological traps is essential, especially given the rapidly increasing number, expanse and built density of urbanized areas, and their associated artificial lighting, around the world [92], [93], [94].

## Supporting Information

Table S1 List of moth species used in the data analyses and their conservation statuses based on Conrad et al. [13]. I = increasing, D = declining, V = vulnerable, NA = not included in the analysis of Conrad et al. [13].
(DOCX)
