# Supplementary material for: Garden and Landscape-Scale Correlates of Moths of Differing Conservation Status: Significant Effects of Urbanization and Habitat Diversity
Source: PLoS One. 2014 Jan 27;9(1):e86925. doi: 10.1371/journal.pone.0086925 (PMC3903603; doi:10.1371/journal.pone.0086925)
Supplement: Table S1 — List of moth species used in the data analyses and their conservation statuses based on Conrad et al. [13]. I = increasing, D = declining, V = vulnerable, NA = not included in the analysis of Conrad et al. [13]. (DOCX) [file pone.0086925.s001.docx]

| **Scientific name** | **Common English name** | **Family** | **Conservation status** |
| --- | --- | --- | --- |
| *Hepialus humuli* (Linnaeus, 1758) | Ghost Moth | Hepialidae | V |
| *Hepialus sylvina* (Linnaeus, 1761) | Orange Swift | Hepialidae | I |
| *Hepialus lupulinus* (Linnaeus, 1758) | Common Swift | Hepialidae | D |
| *Plutella xylostella* (Linnaeus, 1758) | Diamond-back Moth | Yponomeutidae | NA |
| *Hofmannophila pseudospretella* (Stainton, 1849) | Brown House-moth | Oecophoridae | NA |
| *Endrosis sarcitrella* (Linnaeus, 1758) | White-shouldered House-moth | Oecophoridae | NA |
| *Tachystola acroxantha* (Meyrick, 1885) | Ruddy Streak | Oecophoridae | NA |
| *Carcina quercana* (Fabricius, 1775) | Oak Longhorned | Oecophoridae | NA |
| *Epiphyas postvittana* (Walker, 1863) | Light Brown Apple Moth | Torticidae | NA |
| *Tortrix viridana* (Linnaeus, 1758) | Green Oak Tortrix | Torticidae | NA |
| *Acleris variegana* ([Denis & Schiffermüller], 1775) | Garden Rose Tortrix | Torticidae | NA |
| *Alucita hexadactyla* Linnaeus, 1758 | Twenty-plume Moth | Alucitidae | NA |
| *Chrysoteuchia culmella* (Linnaeus, 1758) | Garden Grass-veneer | Crambidae | NA |
| *Agriphila straminella* ([Denis & Schiffermüller], 1775) | Agriphila straminella | Crambidae | NA |
| *Agriphila tristella* ([Denis & Schiffermüller], 1775) | Agriphila tristella | Crambidae | NA |
| *Elophila nymphaeata* (Linnaeus, 1758) | Brown China-mark | Crambidae | NA |
| *Evergestis forficalis* (Linnaeus, 1758) | Garden Pebble | Crambidae | NA |
| *Eurrhypara hortulata* (Linnaeus, 1758) | Small Magpie | Crambidae | NA |
| *Phlyctaenia coronata* (Hufnagel, 1767) | Spotted Magpie | Crambidae | NA |
| *Udea olivalis* ([Denis & Schiffermüller], 1775) | Udea olivalis | Crambidae | NA |
| *Udea ferrugalis* (Hübner, 1796) | Rusty Dot Pearl | Crambidae | NA |
| *Nomophila noctuella* ([Denis & Schiffermüller], 1775) | Rush Veneer | Crambidae | NA |
| *Pleuroptya ruralis* (Scopoli, 1763) | Mother of Pearl | Crambidae | NA |
| *Hypsopygia costalis* (Fabricius, 1775) | Gold Triangle | Pyralidae | NA |
| *Aphomia sociella* (Linnaeus, 1758) | Bee Moth | Pyralidae | NA |
| *Cilix glaucata* (Scopoli, 1763) | Chinese Character | Drepanidae | D |
| *Thyatira batis* (Linnaeus, 1758) | Peach Blossom | Thyatiridae | D |
| *Habrosyne pyritoides* (Hufnagel, 1766) | Buff Arches | Thyatiridae | D |
| *Alsophila aescularia* ([Denis & Schiffermüller], 1775) | March Moth | Geometridae | D |
| *Hemithea aestivaria* (Hübner, 1799) | Common Emerald | Geometridae | D |
| *Timandra comae* (Schmidt, 1931) | Blood-vein | Geometridae | V |
| *Scopula imitaria* (Hübner, 1799) | Small Blood-vein | Geometridae | D |
| *Idaea rusticata* Lempke, 1967 | Least Carpet | Geometridae | NA |
| *Idaea biselata* (Hufnagel, 1767) | Small Fan-footed Wave | Geometridae | D |
| *Idaea seriata* (Schrank, 1802) | Small Dusty Wave | Geometridae | I |
| *Idaea dimidiata* (Hufnagel, 1767) | Single-dotted Wave | Geometridae | I |
| *Idaea aversata* (Linnaeus, 1758) | Riband Wave | Geometridae | I |
| *Xanthorhoe designata* (Hufnagel, 1767) | Flame Carpet | Geometridae | I |
| *Xanthorhoe montanata* ([Denis & Schiffermüller], 1775) | Silver-ground Carpet | Geometridae | D |
| *Xanthorhoe fluctuata* (Linnaeus, 1758) | Garden Carpet | Geometridae | D |
| *Scotopteryx chenopodiata* (Linnaeus, 1758) | Shaded Broad-bar | Geometridae | V |
| *Epirrhoe alternata* (Müller, 1764) | Common Carpet | Geometridae | D |
| *Camptogramma bilineata* (Linnaeus, 1758) | Yellow Shell | Geometridae | I |
| *Anticlea badiata* ([Denis & Schiffermüller], 1775) | Shoulder-stripe | Geometridae | D |
| *Anticlea derivata* ([Denis & Schiffermüller], 1775) | The Streamer | Geometridae | D |
| *Eulithis pyraliata* ([Denis & Schiffermüller], 1775) | Barred Straw | Geometridae | D |
| *Ecliptopera silaceata* ([Denis & Schiffermüller], 1775) | Small Phoenix | Geometridae | V |
| *Chloroclysta siterata* (Hufnagel, 1767) | Red-green Carpet | Geometridae | I |
| *Chloroclysta truncata* (Hufnagel, 1767) | Common Marbled Carpet | Geometridae | D |
| *Cidaria fulvata* (Forster, 1771) | Barred Yellow | Geometridae | D |
| *Thera obeliscata* (Hübner, 1787) | Grey Pine Carpet | Geometridae | I |
| *Thera britannica* (Turner H J, 1925) | Spruce Carpet | Geometridae | I |
| *Colostygia pectinataria* (Knoch, 1781) | Green Carpet | Geometridae | I |
| *Hydriomena furcata* (Thunberg, 1784) | July Highflyer | Geometridae | I |
| *Epirrita dilutata* ([Denis & Schiffermüller], 1775), *christyi* (Allen, 1906), *autumnata* (Borkhausen, 1794) | November Moth agg. | Geometridae | NA |
| *Perizoma affinitata* (Stephens, 1831) | The Rivulet | Geometridae | D |
| *Perizoma alchemillata* (Linnaeus, 1758) | Small Rivulet | Geometridae | D |
| *Eupithecia centaureata* ([Denis & Schiffermüller], 1775) | Lime-speck Pug | Geometridae | NA |
| *Pasiphila rectangulata* (Linnaeus, 1758) | Green Pug | Geometridae | NA |
| *Gymnoscelis rufifasciata* (Haworth, 1809) | Double-striped Pug | Geometridae | NA |
| *Abraxas grossulariata* (Linnaeus, 1758) | The Magpie | Geometridae | D |
| *Lomaspilis marginata* (Linnaeus, 1758) | Clouded Border | Geometridae | D |
| *Macaria liturata* (Clerck, 1759) | Tawny-barred Angle | Geometridae | I |
| *Petrophora chlorosata* (Scopoli, 1763) | Brown Silver-line | Geometridae | D |
| *Opisthograptis luteolata* (Linnaeus, 1758) | Brimstone Moth | Geometridae | D |
| *Ennomos alniaria* (Linnaeus, 1758) | Canary-shouldered Thorn | Geometridae | D |
| *Selenia dentaria* (Fabricius, 1775) | Early Thorn | Geometridae | D |
| *Selenia tetralunaria* (Hufnagel, 1767) | Purple Thorn | Geometridae | D |
| *Odontopera bidentata* (Clerck, 1759) | Scalloped Hazel | Geometridae | D |
| *Crocallis elinguaria* (Linnaeus, 1758) | Scalloped Oak | Geometridae | D |
| *Ourapteryx sambucaria* (Linnaeus, 1758) | Swallow-tailed Moth | Geometridae | D |
| *Colotois pennaria* (Linnaeus, 1761) | Feathered Thorn | Geometridae | D |
| *Lycia hirtaria* (Clerck, 1759) | Brindled Beauty | Geometridae | V |
| *Biston strataria* (Hufnagel, 1767) | Oak Beauty | Geometridae | D |
| *Biston betularia* (Linnaeus, 1758) | Peppered Moth | Geometridae | D |
| *Agriopis marginaria* (Fabricius, 1777) | Dotted Border | Geometridae | D |
| *Peribatodes rhomboidaria* ([Denis & Schiffermüller], 1775) | Willow Beauty | Geometridae | D |
| *Alcis repandata* (Linnaeus, 1758) | Mottled Beauty | Geometridae | I |
| *Cabera pusaria* (Linnaeus, 1758) | Common White Wave | Geometridae | I |
| *Cabera exanthemata* (Scopoli, 1763) | Common Wave | Geometridae | I |
| *Lomographa temerata* ([Denis & Schiffermüller], 1775) | Clouded Silver | Geometridae | D |
| *Campaea margaritata* (Linnaeus, 1767) | Light Emerald | Geometridae | I |
| *Mimas tiliae* (Linnaeus, 1758) | Lime Hawk-moth | Sphingidae | NA |
| *Laothoe populi* (Linnaeus, 1758) | Poplar Hawk-moth | Sphingidae | D |
| *Deilephila elpenor* (Linnaeus, 1758) | Elephant Hawk-moth | Sphingidae | NA |
| *Phalera bucephala* (Linnaeus, 1758) | Buff-tip | Notodontidae | D |
| *Notodonta dromedarius* (Linnaeus, 1767) | Iron Prominent | Notodontidae | D |
| *Notodonta ziczac* (Linnaeus, 1758) | Pebble Prominent | Notodontidae | D |
| *Pheosia gnoma* (Fabricius, 1777) | Lesser Swallow Prominent | Notodontidae | D |
| *Pheosia tremula* (Clerck, 1759) | Swallow Prominent | Notodontidae | I |
| *Ptilodon capucina* (Linnaeus, 1758) | Coxcomb Prominent | Notodontidae | D |
| *Pterostoma palpina* (Clerck, 1759) | Pale Prominent | Notodontidae | D |
| *Drymonia ruficornis* (Hufnagel, 1766) | Lunar Marbled Brown | Notodontidae | I |
| *Orgyia antiqua* (Linnaeus, 1758) | The Vapourer | Lymantriidae | NA |
| *Calliteara pudibunda* (Linnaeus, 1758) | Pale Tussock | Lymantriidae | D |
| *Euproctis similis* (Fuessly, 1775) | Yellow-tail | Lymantriidae | D |
| *Eilema griseola* (Hübner, 1803) | Dingy Footman | Arctiidae | I |
| *Eilema lurideola* (Zincken, 1817) | Common Footman | Arctiidae | I |
| *Arctia caja* (Linnaeus, 1758) | Garden Tiger | Arctiidae | V |
| *Spilosoma lubricipeda* (Linnaeus, 1758) | White Ermine | Arctiidae | V |
| *Spilosoma luteum* (Hufnagel, 1766) | Buff Ermine | Arctiidae | V |
| *Diaphora mendica* (Clerck, 1759) | Muslin Moth | Arctiidae | I |
| *Phragmatobia fuliginosa* (Linnaeus, 1758) | Ruby Tiger | Arctiidae | I |
| *Nola cucullatella* (Linnaeus, 1758) | Short-cloaked Moth | Nolidae | D |
| *Agrotis segetum* ([Denis & Schiffermüller], 1775) | Turnip Moth | Noctuidae | D |
| *Agrotis exclamationis* (Linnaeus, 1758) | Heart & Dart | Noctuidae | D |
| *Agrotis ipsilon* (Hufnagel, 1766) | Dark Sword-grass | Noctuidae | D |
| *Agrotis puta* (Hübner, 1803) | Shuttle-shaped Dart | Noctuidae | I |
| *Axylia putris* (Linnaeus, 1761) | The Flame | Noctuidae | D |
| *Ochropleura plecta* (Linnaeus, 1761) | Flame Shoulder | Noctuidae | D |
| *Noctua pronuba* (Linnaeus, 1758) | Large Yellow Underwing | Noctuidae | I |
| *Noctua comes* Hübner, 1813 | Lesser Yellow Underwing | Noctuidae | I |
| *Noctua fimbriata* (Schreber, 1759) | Broad-bordered Yellow Underwing | Noctuidae | I |
| *Noctua janthe* (Borkhausen, 1792) | Lesser Broad-bordered Yellow Underwing | Noctuidae | I |
| *Noctua interjecta* Schawerda, 1919 | Least Yellow Underwing | Noctuidae | NA |
| *Lycophotia porphyrea* ([Denis & Schiffermüller], 1775) | True Lover's Knot | Noctuidae | D |
| *Diarsia mendica* (Fabricius, 1775) | Ingrailed Clay | Noctuidae | D |
| *Diarsia rubi* (Vieweg, 1790) | Small Square-spot | Noctuidae | V |
| *Xestia c-nigrum* (Linnaeus, 1758) | Setaceous Hebrew Character | Noctuidae | I |
| *Xestia triangulum* (Hufnagel, 1766) | Double Square-spot | Noctuidae | D |
| *Xestia baja* ([Denis & Schiffermüller], 1775) | Dotted Clay | Noctuidae | D |
| *Xestia sexstrigata* (Haworth, 1809) | Six-striped Rustic | Noctuidae | D |
| *Xestia xanthographa* ([Denis & Schiffermüller], 1775) | Square-spot Rustic | Noctuidae | I |
| *Naenia typica* (Linnaeus, 1758) | The Gothic | Noctuidae | D |
| *Mamestra brassicae* (Linnaeus, 1758) | Cabbage Moth | Noctuidae | D |
| *Melanchra persicariae* (Linnaeus, 1761) | Dot Moth | Noctuidae | V |
| *Lacanobia thalassina* (Hufnagel, 1766) | Pale-shouldered Brocade | Noctuidae | I |
| *Lacanobia oleracea* (Linnaeus, 1758) | Bright-line Brown-eye | Noctuidae | D |
| *Melanchra pisi* (Linnaeus, 1758) | Broom Moth | Noctuidae | NA |
| *Orthosia cruda* ([Denis & Schiffermüller], 1775) | Small Quaker | Noctuidae | I |
| *Orthosia gracilis* ([Denis & Schiffermüller], 1775) | Powdered Quaker | Noctuidae | V |
| *Orthosia cerasi* (Fabricius, 1775) | Common Quaker | Noctuidae | I |
| *Orthosia incerta* (Hufnagel, 1766) | Clouded Drab | Noctuidae | D |
| *Orthosia munda* ([Denis & Schiffermüller], 1775) | Twin-spotted Quaker | Noctuidae | D |
| *Orthosia gothica* (Linnaeus, 1758) | Hebrew Character | Noctuidae | D |
| *Mythimna conigera* ([Denis & Schiffermüller], 1775) | Brown-line Bright-eye | Noctuidae | D |
| *Mythimna ferrago* (Fabricius, 1787) | The Clay | Noctuidae | D |
| *Mythimna impura* (Hübner, 1808) | Smoky Wainscot | Noctuidae | D |
| *Mythimna pallens* (Linnaeus, 1758) | Common Wainscot | Noctuidae | D |
| *Mythimna comma* (Linnaeus, 1761) | Shoulder-striped Wainscot | Noctuidae | V |
| *Aporophyla nigra* (Haworth, 1809) | Black Rustic | Noctuidae | D |
| *Lithophane ornitopus* (Dadd, 1911) | Grey Shoulder-knot | Noctuidae | I |
| *Lithophane leautieri* Boursin, 1957 | Blair's Shoulder-knot | Noctuidae | I |
| *Xylocampa areola* (Esper, 1789) | Early Grey | Noctuidae | I |
| *Allophyes oxyacanthae* (Linnaeus, 1758) | Green-brindled Crescent | Noctuidae | NA |
| *Dryobotodes eremita* (Fabricius, 1775) | Brindled Green | Noctuidae | I |
| *Eupsilia transversa* (Hufnagel, 1766) | The Satellite | Noctuidae | I |
| *Conistra vaccinii* (Linnaeus, 1761) | The Chestnut | Noctuidae | I |
| *Conistra ligula* (Esper, 1791) | Dark Chestnut | Noctuidae | D |
| *Agrochola circellaris* (Hufnagel, 1766) | The Brick | Noctuidae | D |
| *Agrochola lota* (Clerck, 1759) | Red-line Quaker | Noctuidae | I |
| *Agrochola macilenta* (Hübner, 1809) | Yellow-line Quaker | Noctuidae | I |
| *Agrochola litura* (Linnaeus, 1761) | Brown-spot Pinion | Noctuidae | V |
| *Agrochola lychnidis* ([Denis & Schiffermüller], 1775) | Beaded Chestnut | Noctuidae | V |
| *Atethmia centrago* (Haworth, 1809) | Centre-barred Sallow | Noctuidae | V |
| *Omphaloscelis lunosa* (Haworth, 1809) | Lunar Underwing | Noctuidae | D |
| *Xanthia aurago* ([Denis & Schiffermüller], 1775) | Barred Sallow | Noctuidae | D |
| *Xanthia togata* (Esper, 1788) | Pink-barred Sallow | Noctuidae | D |
| *Xanthia icteritia* (Hufnagel, 1766) | The Sallow | Noctuidae | V |
| *Acronicta aceris* (Linnaeus, 1758) | The Sycamore | Noctuidae | NA |
| *Acronicta leporina* (Linnaeus, 1758) | The Miller | Noctuidae | NA |
| *Acronicta psi* (Linnaeus, 1758), *tridens* ([Denis & Schiffermüller], 1775) | Grey Dagger agg. | Noctuidae | NA |
| *Acronicta rumicis* (Linnaeus, 1758) | Knot Grass | Noctuidae | V |
| *Cryphia domestica* (Hufnagel, 1766) | Marbled Beauty | Noctuidae | I |
| *Amphipyra pyramidea* (Linnaeus, 1758), *berbera* Fletcher, 1968 | Copper Underwing agg. | Noctuidae | NA |
| *Amphipyra tragopoginis* (Clerck, 1759) | Mouse Moth | Noctuidae | V |
| *Rusina ferruginea* (Esper, 1758) | Brown Rustic | Noctuidae | D |
| *Euplexia lucipara* (Linnaeus, 1758) | Small Angle Shades | Noctuidae | D |
| *Phlogophora meticulosa* (Linnaeus, 1758) | Angle Shades | Noctuidae | I |
| *Cosmia trapezina* (Linnaeus, 1758) | The Dun-bar | Noctuidae | D |
| *Apamea monoglypha* (Hufnagel, 1766) | Dark Arches | Noctuidae | D |
| *Apamea lithoxylaea* ([Denis & Schiffermüller], 1775) | Light Arches | Noctuidae | D |
| *Apamea crenata* (Hufnagel, 1766) | Clouded-bordered Brindle | Noctuidae | D |
| *Apamea remissa* (Hübner, 1809) | Dusky Brocade | Noctuidae | V |
| *Apamea sordens* (Hufnagel, 1766) | Rustic Shoulder-knot | Noctuidae | D |
| *Oligia strigilis* (Linnaeus, 1758), *versicolor* (Borkhausen, 1792), *latruncula* ([Denis & Schiffermüller], 1775) | Marbled Minor agg. | Noctuidae | NA |
| *Oligia fasciuncula* (Haworth, 1809) | Middle-barred Minor | Noctuidae | D |
| *Mesapamea secalis* (Linnaeus, 1758), *didyma* (Espa, 1788), *remmi* Rezbanyai-Reser, 1985 | Common Rustic agg. | Noctuidae | NA |
| *Luperina testacea* ([Denis & Schiffermüller], 1775) | Flounced Rustic | Noctuidae | D |
| *Hydraecia micacea* (Espa, 1789) | Rosy Rustic | Noctuidae | V |
| *Gortyna flavago* ([Denis & Schiffermüller], 1775) | Frosted Orange | Noctuidae | D |
| *Hoplodrina ambigua* ([Denis & Schiffermüller], 1775) | Vine's Rustic | Noctuidae | I |
| *Caradrina morpheus* (Hufnagel, 1766) | Mottled Rustic | Noctuidae | V |
| *Paradrina clavipalpis* (Scopoli, 1763) | Pale Mottled Willow | Noctuidae | I |
| *Diachrysia chrysitis* (Linnaeus, 1758) | Burnished Brass | Noctuidae | D |
| *Polychrysia moneta* (Fabricius, 1787) | Golden Plusia | Noctuidae | NA |
| *Autographa gamma* (Linnaeus, 1758) | Silver Y | Noctuidae | D |
| *Autographa pulchrina* (Haworth, 1809) | Beautiful Golden Y | Noctuidae | D |
| *Autographa jota* (Linnaeus, 1758) | Plain Golden Y | Noctuidae | D |
| *Abrostola tripartita* (Hufnagel, 1766) | The Spectacle | Noctuidae | I |
| *Scoliopteryx libatrix* (Linnaeus, 1758) | The Herald | Noctuidae | NA |
| *Rivula sericealis* (Scopoli, 1763) | Straw Dot | Noctuidae | I |
| *Hypena proboscidalis* (Linnaeus, 1758) | The Snout | Noctuidae | D |
| *Zanclognatha tarsipennalis* (Treitschke, 1835) | The Fan-foot | Noctuidae | D |
| *Herminia grisealis* ([Denis & Schiffermüller], 1775) | Small Fan-foot | Noctuidae | D |
